# Physical Activity and Lung Function in Adolescents: The 1993 Pelotas (Brazil) Birth Cohort Study

**DOI:** 10.1016/j.jadohealth.2012.06.023

**Published:** 2012-12

**Authors:** Ana M.B. Menezes, Fernando César Wehrmeister, Ludmila Correa Muniz, Rogelio Perez-Padilla, Ricardo B. Noal, Marcelo C. Silva, Helen Gonçalves, Pedro C. Hallal

**Affiliations:** a Postgraduate Program in Epidemiology, Federal University of Pelotas, Pelotas, Brazil; b Institute of Respiratory Diseases, México City, Mexico

**Keywords:** Physical activity, Lung function, Cohort, Adolescents

## Abstract

**Purpose:**

To evaluate the association between physical activity changes in those aged 11 to 15 years and lung function at age 15 years.

**Methods:**

The original cohort comprised 5,249 hospital-born children during the calendar year of 1993 in Pelotas, Brazil. In 2004–2005 and 2008–2009, all cohort members were sought for follow-up visits. Self-reported physical activity was measured at ages 11 and 15 years. At the 2008–2009 visit, when participants were 15 years old, spirometry was performed. Linear regression was used, and all analyses were stratified by sex.

**Results:**

Of the 5,249 original members of the cohort, 4,325 were located at 15 years of age, and spirometry was performed on 4,010 members. Forced expiratory volume in 1 second was not associated with physical activity. In girls, those who were active in leisure time in both periods have better percent-predicted forced vital capacity (*β* = 3.573 [95% confidence interval {CI}: 1.015, 6.130]) and forced expiratory volume in the 6 seconds (*β* = .095 [95% CI: .021, .168]) than those who were inactive in the two periods. Also in girls, those who became active at 15 years of age had higher peak expiratory flow than those who were inactive at 11 and 15 years of age. In boys, only those who became inactive in leisure time had worse peak expiratory flow (*β* = −.180 [95% CI: −.339, −.021]) than boys who were inactive at ages 11 and 15 years.

**Conclusions:**

Self-reported leisure-time physical activity was associated with better effort-dependent lung function parameters, particularly among girls.

**Implications and Contribution**

Adolescent girls were more likely to have better parameters of lung function at 15 years of age if they were active at age 11 and 15 years, particularly in comparison with those who were inactive in the two periods. Girls who became active at 15 years of age had also higher parameters of lung function such as PEF than those inactive at 11 and 15 years of age.

Global public health priorities have been shifting in recent decades. Contributing factors include the aging of the population, rapid urbanization, industrialization, and globalization, among others. The combination of these factors has led to an increase in unhealthy lifestyles [1]. Physical inactivity is directly related to morbidity and mortality from several diseases [2], and its promotion has been recently considered a priority by the United Nations [3]. Physical activity in childhood and adolescence provides a series of short- and long-term benefits for health and well-being [1].

In a literature review, Lucas and Platts-Mills [4] showed an inverse association between physical activity practice and respiratory health, particularly asthma. However, there is no consensus in the literature regarding this association. Another systematic review found no evidence that physical activity was related to resting lung function or number of asthma crises [5]. Finally, another review focused exclusively on swimming concluded that its practice was inversely related to the number of asthma crises [6]. It should be mentioned that subjects with asthma do not necessarily have abnormalities in lung function in the absence of the asthma crisis.

Part of the inconsistency observed in the literature is owing to the plethora of cross-sectional studies [7–9]. One of the few prospective studies, using data from the Avon Longitudinal Study of Parents and Children (ALSPAC) cohort in the United Kingdom [10], found higher lung function values (forced expiratory volume in the first second [FEV_1_] and forced vital capacity [FVC]) among 8-year-old children who used to swim at the ages of 4 and 7 years.

Both physical activity and lung function have been shown to track from adolescence to adulthood [11,12]. The aim of the present study was to evaluate the association between physical activity changes in children from ages 11 to 15 years and lung function at age 15 years in a birth cohort study from Brazil.

## Methods

Hospital-born children in the city of Pelotas, Brazil, in the calendar year of 1993 (>99% of all deliveries) are part of a birth cohort study. Of 5,265 mothers who gave birth that year, 5,249 agreed to take part. In 2004–2005 and 2008–2009, all cohort members were sought for follow-up visits, and questionnaires were administered by trained interviewers at the participant's household. At the 2008–2009 visit, when participants were, on average, 15 years old, maximally forced expiratory volume maneuvers were performed. Methodological details about the spirometric tests are available elsewhere [13,14].

In summary, adolescents performed the tests while they were seated, with a nose-clip and a disposable 500-mL spacer. A battery-operated portable spirometer was used (Easy-One2; NDD Medical Technologies, Chelmsford, MA and Zurich, Switzerland). Standard exclusion criteria were used, including thoracic, eye, or abdominal surgery within the previous 3 months; heart conditions; or hospital admissions in the previous 3 months. We also excluded pregnant girls and those who reported being in treatment for tuberculosis. Based on all these criteria, 64 adolescents were not eligible for spirometry. The mean of seven maneuvers was performed by the adolescents; 12% of them were asked to perform more tests (up to 15) to produce three acceptable curves [15]. Approximately 90% of the spirometric tests reached quality grade A, that is, a reproducibility of FEV_1_ and FVC to 150 mL (91.2% pre-bronchodilator [BD] and 90.0% post-BD). Outcome variables included FVC and FEV_1_, both expressed as percentage of predicted value [16]. We also evaluate forced expiratory volume in 6 seconds (FEV_6_) and the peak expiratory flow (PEF).

Physical activity was assessed through self-report, both at 11 and 15 years of age. The questionnaire covered practice of leisure-time physical activity, including sports practice inside and outside school, and commuting physical activity. According to current global physical activity guidelines, a cutoff point of 300 min/wk was used to classify adolescents as physically active or not [1]. Physical activity changes were constructed separately for leisure-time and total physical activity by categorizing adolescents as inactive–inactive (did not reach the 300 min/wk cutoff point at age 11 or 15 years); inactive–active (reached the threshold at the age 15-year visit only); active–inactive (reached the threshold at the age 11-year visit only); or active–active (reached the threshold in both visits). The reliability and validity of this questionnaire were tested in a sample of adolescents from the same city. The questionnaire presented high reliability scores and moderate concurrent validity using pedometers as the reference method [17].

Confounding variables included family income, maternal schooling, birth weight, maternal smoking during pregnancy, maternal height, adolescent height, wheezing in past year, allergy status, body mass index, and asthma medication past 2 weeks. Because the association between adolescent height and lung function is known to be nonlinear, we used both quadratic and logarithm transformation in the models. Results were similar, and therefore only the latter are presented. Following statistically significant *p* values showing interaction between sex and physical activity at predicting lung function, all analyses were stratified by sex. Data were analyzed using Stata 11.0 (StataCorp, College Station, TX).

All phases of the 1993 Pelotas (Brazil) Birth Cohort Study obtained approval from certified institutional review boards. Parents or guardians provided written informed consent in all follow-up visits, and adolescents provided verbal consent.

## Results

Of the 5,249 original members of the cohort, 4,325 were located at 15 years of age, corresponding to a total follow-up rate of 85.7%, taking into account those known to have died in the period. Of the 4,325 adolescents located, we obtained valid spirometric data for 4,010 (51% girls).

Table 1 describes physical activity changes from 11 to 15 years of age, lung function at 15 years by sex, and the covariates included in the analyses. Around one in every three mothers reported to smoke during pregnancy. About one in every four had nine or more years of formal education. The proportion of low birth weight was 8.7% among boys and 10.8% among girls. Eleven percent of the boys and 13.2% reported wheezing in the past year. Only .5% of the adolescents used asthma medication in the past 2 weeks. Around one in every four reported to have a diagnosis of allergy.

Among boys, 20% were inactive in both follow-up visits, whereas the equivalent proportion among girls was 46%. On the other hand, 38% of the boys and only 15% of the girls were active in both follow-up waves. In terms of leisure-time physical activity only, 30% of the boys and 61% of the girls were inactive at both time points. Also, 26% of the boys and only 7% of the girls were active in both visits. Mean pre-BD FEV_1_ was .53 L higher in boys than girls. Mean pre-BD FVC was .71 L higher among boys than girls. In terms of percentage of predicted values, the mean FEV_1_ was 95.6 among boys and 107.9 among girls, whereas mean FVC was 103.2 among boys and 108.4 among girls. FEV_6_ and PEF were also higher among boys than girls.

In Table 2, we present unadjusted results of the association between physical activity changes and lung function parameters among girls. Both in terms of leisure-time and total physical activity, the only statistically significant findings were in relation to PEF. Those who became active at 15 years had higher average PEF values than those who continued to be inactive. This finding was confirmed in the adjusted analyses (Table 3). In addition, those who were active in both time points had higher average FVC and FEV_6_ values as compared with those who were inactive at 11 and 15 years. FEV_1_ was reduced among girls who were inactive and became active, as well for those who were active and became inactive from 11 to 15 years. Girls who were categorized as active in terms of leisure-time physical activity, at both ages, had an increase in FEV_1_, but it was not statistically significant. These findings were nearly the same in the unadjusted and adjusted analysis (Tables 2 and 3).

Findings for leisure-time and total physical activity in boys are displayed in Tables 4 and 5. Both in the unadjusted and in the adjusted analyses, boys who became inactive from age 11 to 15 years had lower PEF values as compared with the reference category. All other associations were nonsignificant.

## Discussion

The literature is inconsistent regarding the association between physical activity and lung function [5,7,18]. Two main problems are detected in the literature on this topic. First, there are still few studies, and virtually none are from low- and middle-income countries. Second, most studies available used cross-sectional designs, which are inappropriate to establish temporality. There is considerable evidence linking physical activity, particularly swimming, with asthma [4,10,19], but only 3.4% of our cohort members reported to swim at 15 years of age, particularly because public swimming pools are extremely rare in most Brazilian cities, and none are available in the city where this study took place. This suggests that the public health impact of swimming is likely to be minimal.

In our study, we prospectively evaluated the association between physical activity changes from age 11 to 15 years and lung function at age 15 years. Among girls, we found that those who became active in the period had benefits in terms of PEF, whereas those who were consistently active had benefits in terms of FVC and FEV_6_. Among boys, findings are similar in terms of the direction of the association, but more intriguing. Those who became inactive in the period had lower PEF values as compared with those who were inactive in both time points. In addition to these statistically significant findings, several other associations were in the same direction but did not reach statistical significance.

The fact that physical activity in our cohort was related to PEF, FEV_6_, and FVC is not surprising. These lung function parameters are effort dependent, as opposed to FEV_1_ (which is effort independent), and therefore a better clinical measure that indicates obstruction [16,20]. Another expected finding is the stronger effect of physical activity on lung function among girls than boys. In the same cohort, we previously showed that 49.2% and 29.5% of lung function variability among boys and girls, respectively, is explained by height and socioeconomic status alone. This suggests that other variables than these two are more likely to influence girls' than boys' lung function [21].

Leisure-time physical activity was generally more strongly associated with lung function parameters as compared with total physical activity. This is also expected. We have previously found that commuting physical activity in this cohort is highly associated with socioeconomic status [22], whereas total physical activity is not. In addition, the intensity of leisure-time physical activity is typically higher than that of commuting physical activity [23].

Comparing our data with previous studies is challenging for several reasons. First, the prospective design used is rare. Second, measures of physical activity used in the literature are highly inconsistent [7,9,10]. Our study also has some strengths that are worth mentioning. More than 90% of the spirometric tests reached American Thoracic Society (ATS) quality standards [24]. Also, we achieved high follow-up rates, minimizing the likelihood of selection bias. Finally, testing this association in a middle-income setting is rare. Some limitations should also be considered. First, we relied on self-reported data on physical activity, which can lead to some degree of misclassification. Second, the observational nature of our study impedes us from evaluating causality in further detail; this could be achieved by conducting randomized trials in this area. Available systematic reviews of clinical studies, mostly with asthmatic patients, showed benefits of exercise on maximum oxygen uptake and heart rate [5], although most studies have small sample sizes. Despite the longitudinal design of the study, the lack of measurement of lung function at age 11 years does not allow us to rule out the possibility of reverse causality; it is possible that lung function problems starting between 11 and 15 years of age may have affected the levels of physical activity in the period. Another limitation is the use of self-reported physical activity instead of objective measurements, such as accelerometry. However, this is likely to attenuate any possible association between physical activity and lung function instead of exacerbating it.

A longitudinal study from The Netherlands with individuals aged 13–27 years found that physical activity was inversely related to PEF but directly related to FVC [25]. In our cohort, positive associations between physical activity and both lung function indicators were found in girls. A study in Tunisia with children aged 6–16 years found no association between physical activity and absolute values of FEV_1_ or FVC [18]. In Norway, among children aged 9 and 10 years, physical activity was related to higher FEV_1_ and FVC in girls but not in boys [7].

A possible relationship between physical activity and lung function might be explained by environmental, behavioral, and genetic factors [26]. Several variables influence the patency of bronchioles [4]: excess mucus, edema caused by poor mucociliary clearance, increased airway smooth muscle, collagen deposition from chronic inflammation, decreased chest wall force, among other factors. The key component of acute airway narrowing is constriction of airway smooth muscle. Decreased airway resistance after deep inspiration in asthmatic and in nonasthmatic subjects was demonstrated [27]. Besides this, significantly lower sigh rates in asthmatic and nonasthmatic subjects while watching a video than while reading was also demonstrated. The combination of decreased exercise coupled with increased hours in front of a screen could contribute to decreased deep inspiration [28]. Linking exercise to lung function is thus plausible.

In summary, we found that physical activity in adolescence is related to improved lung function, particularly among girls. Both physical activity and lung function are related to noncommunicable diseases, which represent the largest share of the burden of disease in the world [29]. Therefore, physical activity promotion strategies are urgently needed to improve lung function and health in general.

## Figures and Tables

**Table 1:** Description of the sample, physical activity changes (age 11–15 years), and lung function parameters at age 15 years, by sex. Pelotas 1993 Birth Cohort

Variable	Male n (%)	Female n (%)
Smoking during pregnancy		
No	1,763 (67.7)	1,733 (65.6)
Yes	843 (32.3)	909 (34.4)
Maternal schooling (years of formal education)		
≥9 years	684 (26.3)	666 (25.2)
5–8 years	1,214 (46.6)	1,210 (45.8)
1–4 years	646 (24.8)	691 (26.2)
0	61 (2.3)	73 (2.8)
Birth weight (grams)		
≥2,500	2,372 (91.3)	2,350 (89.2)
<2,500	226 (8.7)	284 (10.8)
Wheezing in the past year		
No	1,880 (89.0)	1,921 (86.8)
Yes	233 (11.0)	291 (13.2)
Asthma medication in the past 2 weeks		
No	2,118 (99.5)	2,202 (99.5)
Yes	10 (.5)	11 (.5)
Allergy status		
No	1,624 (76.6)	1,612 (73.1)
Yes	496 (23.4)	594 (26.9)
Mother's height at birth, cm (mean [SD])	159.8 (6.7)	159.8 (6.8)
Adolescent's height, cm (mean [SD])	167.2 (8.0)	159.2 (6.3)
Body mass index, kg/cm^2^ (mean [SD])	21.4 (4.0)	21.6 (3.9)
Changes in total PA (11–15 years)		
Inactive–inactive	401 (19.9)	958 (45.9)
Inactive–active	459 (22.8)	361 (17.3)
Active–inactive	379 (18.9)	464 (22.2)
Active–active	772 (38.4)	304 (14.6)
Changes in leisure-time PA (11–15 years)		
Inactive–inactive	601 (29.7)	1,273 (60.7)
Inactive–active	505 (25.0)	294 (14.0)
Active–inactive	387 (19.1)	389 (18.6)
Active–active	531 (26.2)	140 (6.7)
FEV_1_ pre-BD, L (mean [±SD])	3.46 (.66)	2.93 (.44)
FVC pre-BD, L (mean [±SD])	4.01 (.76)	3.30 (.51)
FEV_1_ pre-BD, % predicted (mean [±SD])	95.64 (13.06)	107.93 (12.77)
FVC pre-BD, % predicted (mean [±SD])	103.15 (13.31)	108.41 (13.44)
FEV_6_ pre-BD, L (mean [±SD])	4.00 (.76)	3.29 (.50)
PEF pre-BD, L/s (mean [±SD])	7.47 (1.45)	6.61 (1.04)

PA = Physical activity; FEV_1_ = forced expiratory volume in 1 second; FEV_6_ = forced expiratory volume in 6 seconds; FVC = forced vital capacity; PEF = peak expiratory flow.

**Table 2:** Crude analyses of changes of physical activity and lung function parameters in female subjects. Pelotas 1993 Birth Cohort

Variable	FEV_1_ (% predicted)		FVC (% predicted)		FEV_6_ (L)		PEF (L/s)	
	*β* (95% CI)	*p* value	*β* (95% CI)	*p* value	*β* (95% CI)	*p* value	*β* (95% CI)	*p* value
Changes of total PA (11–15 years)								
Inactive–inactive	Reference		Reference		Reference		Reference	
Inactive–active	−1.183 (−2.810, .445)	.154	−.987 (−2.744, .770)	.271	.036 (−.027, .098)	.267	.127 (−.004, .258)	.058
Active–inactive	−.352 (−1.846, 1.141)	.643	.002 (−1.611, 1.615)	.998	.017 (−.040, .075)	.552	.014 (−.106, .134)	.816
Active–active	.466 (−1.261, 2.193)	.597	.991 (−.874, 2.855)	.298	.054 (−.012, .121)	.111	.093 (−.046, .232)	.188
Changes of leisure-time PA (11–15 years)								
Inactive–inactive	Reference		Reference		Reference		Reference	
Inactive–active	.848 (−.862, 2.558)	.331	.873 (−.971, 2.718)	.353	.097 (.031, .163)	.004	.266 (.129, .403)	<.001
Active–inactive	.597 (−.922, 2.117)	.441	.527 (−1.112, 2.167)	.528	.055 (−.004, .113)	.067	.077 (−.045, .199)	.216
Active–active	1.731 (−.625, 4.088)	.150	3.251 (.709, 5.794)	.012	.113 (.022, .203)	.015	.123 (−.066, .312)	.202

PA = Physical activity; FEV_1_ = forced expiratory volume in 1 second; FEV_6_ = forced expiratory volume in 6 seconds; FVC = forced vital capacity; PEF = peak expiratory flow.

**Table 3:** Adjusted analyses of changes of physical activity and lung function parameters in female subjects. Pelotas 1993 Birth Cohort

Variable	FEV_1_ (% predicted)		FVC (% predicted)		FEV_6_ (L)		PEF (L/s)	
	*β* (95% CI)	*p* value	*β* (95% CI)	*p* value	*β* (95% CI)	*p* value	*β* (95% CI)	*p* value
Changes of total PA (11–15 years)								
Inactive–inactive	Reference		Reference		Reference		Reference	
Inactive–active	−.875 (−2.505, .755)	.293	−1.033 (−2.791, .725)	.249	−.033 (−.084, .017)	.198	.040 (−.082, .162)	.519
Active–inactive	−.117 (−1.620, 1.386)	.879	−.102 (−1.723, 1.518)	.901	−.012 (−.058, .035)	.622	.002 (−.111, .114)	.973
Active–active	1.066 (−.664, 2.797)	.227	1.362 (−.504, 3.228)	.152	.041 (−.012, .095)	.131	.069 (−.061, .198)	.299
Changes of leisure-time PA (11–15 years)								
Inactive–inactive	Reference		Reference		Reference		Reference	
Inactive–active	.960 (−.748, 2.669)	.270	.754 (−1.087, 2.595)	.422	.034 (−.019, .087)	.215	**.178** (**.051, .306**)	**.006**
Active–inactive	.704 (−.823, 2.231)	.366	.435 (−1.210, 2.080)	.604	.018 (−.029, .065)	.457	.043 (−.072, .157)	.465
Active–active	2.172 (−.202, 4.546)	.073	**3.573** (**1.015, 6.130**)	**.006**	**.095** (**.021, .168**)	**.012**	.092 (−.086, .269)	.310

Adjusted for family income at birth, maternal schooling at birth, birth weight, smoking during pregnancy, mother's height at birth, adolescent height at 15 years, wheezing in past year, body mass index, allergy status, and asthma medication. Values in bold indicate statistically significant regression coefficients (*p* <.05).

**Table 4:** Crude analyses of changes of physical activity and lung function parameters in male subjects. Pelotas 1993 Birth Cohort

Variable	FEV_1_ (% predicted)		FVC (% predicted)		FEV_6_ (L)		PEF (L/s)	
	*β* (95% CI)	*p* value	*β* (95% CI)	*p* value	*β* (95% CI)	*p* value	*β* (95% CI)	*p* value
Changes of total PA (11–15 years)								
Inactive–inactive	Reference		Reference		Reference		Reference	
Inactive–active	.668 (−1.174, 2.511)	.477	1.771 (−.133, 3.675)	.068	.032 (−.073, .137)	.552	−.069 (−.270, .132)	.501
Active–inactive	−.853 (−2.776, 1.070)	.384	−.217 (−2.204, 1.770)	.830	.055 (−.055, .164)	.326	−.064 (−.273, .146)	.549
Active–active	−1.143 (−2.799, .512)	.176	−.656 (−2.367, 1.054)	.452	−.066 (−.160, .028)	.168	−.097 (−.278, .083)	.291
Changes of leisure-time PA (11–15 years)								
Inactive–inactive	Reference		Reference		Reference		Reference	
Inactive–active	.272 (−1.353, 1.897)	.743	1.236 (−.443, 2.915)	.149	.022 (−.070, .115)	.634	−.102 (−.279, .075)	.258
Active–inactive	−1.416 (−3.168, .335)	.113	−.888 (−2.698, .923)	.336	−.020 (−.119, .080)	.702	−.205 (−.396, −.015)	.035
Active–active	−.823 (−2.426, .781)	.315	−.490 (−2.148, 1.167)	.562	−.044 (−.135, .048)	.349	−.102 (−.277, .072)	.252

PA = Physical activity; FEV_1_ = forced expiratory volume in 1 second; FEV_6_ = forced expiratory volume in 6 seconds; FVC = forced vital capacity; PEF = peak expiratory flow.

**Table 5:** Adjusted analyses of changes of physical activity and lung function parameters in male subjects. Pelotas 1993 Birth Cohort

Variable	FEV_1_ (% predicted)		FVC (% predicted)		FEV_6_ (L)		PEF (L/s)	
	*β* (95% CI)	*p* value	*β* (95% CI)	*p* value	*β* (95% CI)	*p* value	*β* (95% CI)	*p* value
Changes of total PA (11–15 years)								
Inactive–inactive	Reference		Reference		Reference		Reference	
Inactive–active	.476 (−1.279, 2.231)	.595	1.776 (−.101, 3.653)	.064	.046 (−.025, .117)	.203	−.063 (−.231, 0.1.5)	.462
Active–inactive	−1.184 (−3.016, .648)	.205	−.379 (−2.338, 1.580)	.704	−.009 (−.084, .065)	.802	−.144 (−.319, .031)	.107
Active–active	−1.247 (−2.828, .333)	.122	−.458 (−2.148, 1.232)	.595	−.022 (−.086, .042)	.505	−.040 (−.191, .111)	.604
Changes of leisure-time PA (11–15 years)								
Inactive–inactive	Reference		Reference		Reference		Reference	
Inactive–active	.204 (−1.339, 1.747)	.796	1.269 (−.381, 2.919)	.132	.040 (−.023, .102)	.212	−.079 (−.227, .068)	.292
Active–inactive	−1.223 (−2.886, .439)	.149	−.635 (−2.413, 1.142)	.484	−.015 (−.082, .052)	.668	**−.180** (**−.339, −.021**)	**.027**
Active–active	−.912 (−2.436, .613)	.241	−.395 (−2.025, 1.235)	.635	−.009 (−.071, .053)	.772	−.054 (−.199, .092)	.471

Adjusted for family income at birth, maternal schooling at birth, birth weight, smoking during pregnancy, mother's height at birth, height at 15 years, wheezing in past year, body mass index, allergy status, and asthma medication.

## References

[1] World Health Organization (2010). Global recommendations on physical activity for health.

[2] U. S. Department of Health and Human Services (2010). Healthy people 2020.

[3] United Nations (2011). 2011 High level meeting on prevention and control of non-communicable diseases [General Assembly]. http://www.un.org/en/ga/ncdmeeting2011/.

[4] Lucas S.R., Platts-Mills T.A. (2005). Physical activity and exercise in asthma: Relevance to etiology and treatment. J Allergy Clin Immunol.

[5] Ram F.S., Robinson S.M., Black P.N. (2005). Physical training for asthma. Cochrane Database Syst Rev.

[6] Rosimini C. (2003). Benefits of swim training for children and adolescents with asthma. J Am Acad Nurse Pract.

[7] Berntsen S., Wisløff T., Nafstad P., Nystad W. (2008). Lung function increases with increasing level of physical activity in school children. Pediatr Exerc Sci.

[8] Fuster V., Rebato E., Fernández López J., Rosique J.R. (2008). Physical activity related to forced vital capacity and strength performance in a sample of young males and females. Coll Antropol.

[9] Holmen T.L., Barrett-Connor E., Clausen J. (2002). Physical exercise, sports, and lung function in smoking versus nonsmoking adolescents. Eur Respir J.

[10] Font-Ribera L., Villanueva C.M., Nieuwenhuijsen M.J. (2011). Swimming pool attendance, asthma, allergies, and lung function in the Avon longitudinal study of parents and children cohort. Am J Respir Crit Care Med.

[11] Hallal P.C., Victora C.G., Azevedo M.R., Wells J.C. (2006). Adolescent physical activity and health: A systematic review. Sports Med.

[12] Lawlor D.A., Ebrahim S., Davey Smith G. (2005). Association of birth weight with adult lung function: Findings from the British women's heart and health study and a meta-analysis. Thorax.

[13] Araújo C.L., Menezes A.M., Vieira Mde F. (2010). The 11-year follow-up of the 1993 Pelotas (Brazil) birth cohort study: Methods. Cad Saude Publica.

[14] Victora C.G., Hallal P.C., Araújo C.L. (2008). Cohort profile: The 1993 Pelotas (Brazil) birth cohort study. Int J Epidemiol.

[15] Pérez-Padilla R., Vázquez-García J.C., Márquez M.N. (2008). Spirometry quality-control strategies in a multinational study of the prevalence of chronic obstructive pulmonary disease. Respir Care.

[16] Pereira C.A.C., Lemle A., Algranti E. (1996). I Consenso brasileiro sobre espirometria. J Pneumol.

[17] Bastos J.P., Araujo C.L., Hallal P.C. (2008). Prevalence of insufficient physical activity and associated factors in Brazilian adolescents. J Phys Act Health.

[18] Trabelsi Y., Pariès J., Harrabi I. (2008). Factors affecting the development of lung function in Tunisian children. Am J Hum Biol.

[19] Basaran S., Guler-Uysal F., Ergen N. (2006). Effects of physical exercise on quality of life, exercise capacity and pulmonary function in children with asthma. J Rehabil Med.

[20] Ferguson G.T., Enright P.L., Buist A.S., Higgins M.W. (2000). Office spirometry for lung health assessment in adults: A consensus statement from the national lung health education program. Chest.

[21] Menezes A.M., Dumith S.C., Perez-Padilla R. (2011). Socioeconomic trajectory from birth to adolescence and lung function: Prospective birth cohort study. BMC Public Health.

[22] Hallal P.C., Wells J.C., Reichert F.F. (2006). Early determinants of physical activity in adolescence: Prospective birth cohort study. BMJ.

[23] Florindo A.A., Guimarães V.V., Cesar C.L. (2009). Epidemiology of leisure, transportation, occupational, and household physical activity: Prevalence and associated factors. J Phys Act Health.

[24] American Thoracic Society (1995). Standardization of spirometry, 1994 update. Am J Respir Crit Care Med.

[25] Twisk J.W., Staal B.J., Brinkman M.N. (1998). Tracking of lung function parameters and the longitudinal relationship with lifestyle. Eur Respir J.

[26] Eisenmann J., Katzmarzyk P., Theriault G. (1999). Physical activity and pulmonary function in youth: The Quebec family study. Pediatr Exerc Sci.

[27] Fish J.E., Ankin M.G., Kelly J.F., Peterman V.I. (1981). Regulation of bronchomotor tone by lung inflation in asthmatic and nonasthmatic subjects. J Appl Physiol.

[28] Hark W.T., Thompson W.M., McLaughlin T.E. (2005). Spontaneous sigh rates during sedentary activity: Watching television vs reading. Ann Allergy Asthma Immunol.

[29] Schmidt M.I., Duncan B.B., Azevedo e Silva G. (2011). Chronic non-communicable diseases in Brazil: Burden and current challenges. Lancet.

